# A Brain Metastasis in Search of Its Origin: Occult Primary Non-small Cell Lung Cancer

**DOI:** 10.7759/cureus.117434

**Published:** 2026-10-04

**Authors:** Hanane El Yachioui, Ahmed BenSghier, Soufiane Berhili, Mohamed Moukhlissi, Loubna Mezouar

**Affiliations:** 1 Department of Radiation Oncology, Faculty of Medicine and Pharmacy, Mohammed First University, Oujda, MAR

**Keywords:** case report, gamma knife stereotactic radiosurgery, immunohistochemistry, isolated brain metastasis, occult primary non-small cell lung cancer

## Abstract

We present a case of a 67-year-old man who presented with right-sided hemiparesis and headache and was found to have a solitary left temporoparietal cerebral lesion on MRI, initially favoring a primary brain tumor. Further workup was unremarkable and the patient underwent surgical resection of the cerebral lesion. Histopathology and immunohistochemistry favored a metastatic pulmonary adenocarcinoma, though imaging scans showed no primary lung lesion. Subsequent PET-CT demonstrated hypermetabolic mediastinal, hilar, and supraclavicular lymphadenopathy, consistent with occult primary non-small cell lung cancer. The patient underwent stereotactic gamma knife radiotherapy to the resection bed, followed by systemic chemotherapy with cisplatin and vinorelbine. At the time of writing, two of three planned chemotherapy cycles had been completed, with radiological response assessment scheduled after completion of the treatment. This case adds to the limited literature on occult primary non-small cell lung cancer (NSCLC) and supports early tissue diagnosis as key to appropriate management.

## Introduction

Establishing a diagnosis of non-small cell lung cancer conventionally relies on identifying a primary lung lesion; however, in rare instances, this sequence is reversed, with metastatic disease as the first and only clue to an underlying, radiographically silent tumor. This diagnostic challenge is particularly critical given the fact that lung cancer remains the leading cause of cancer-related mortality worldwide [1]. Non-small cell lung cancer (NSCLC) is the most common histologic category of lung cancer [2].

NSCLC encompasses several histologic subtypes, principally adenocarcinoma, squamous cell carcinoma, and large cell carcinoma [2]. Locally, these tumors may present with cough, hemoptysis, dyspnea, or chest pain. As the disease advances, NSCLC, commonly metastasizes to the liver, brain, bone, adrenal glands, and pericardium [3]. Brain metastases are a clinically significant manifestation of NSCLC, occurring at initial diagnosis in approximately 10% of patients overall and in up to 26% of those presenting with stage IV disease [4]. 

Occult NSCLC is a rare clinical entity. Within the American Joint Committee on Cancer (AJCC) staging framework, occult lung cancer denotes malignant cells detected in sputum or bronchial washings without a radiographically or bronchoscopically visible primary tumor [5]. In this report, the clinically broader term occult primary NSCLC (OP-NSCLC) describes metastatic adenocarcinoma with convincing pulmonary differentiation despite the absence of a radiologically identifiable primary lung lesion.

We report a rare case of OP-NSCLC that initially presented with a brain metastasis and no identifiable primary lung lesion; the diagnosis was established through immunohistochemical analysis of the cerebral metastasis.

## Case presentation

A 67-year-old man with a long-standing history of tobacco and alcohol use and no other notable medical history presented to our hospital with right-sided hemiparesis and headache. Brain MRI revealed an intra-axial, cortico-subcortical lesion in the left temporoparietal region, measuring 21 mm in maximal diameter (Figure 1), with associated mass effect on the ipsilateral lateral ventricle and no midline shift. The process was potentially consistent with oligodendroglioma. The patient underwent en-bloc resection of the lesion; histopathological examination of the resected specimen revealed morphological features consistent with a cerebral metastasis arising from a carcinoma. Immunohistochemical analysis of the resected cerebral lesion demonstrated positivity for AE1/AE3, CK7, thyroid transcription factor 1 (TTF-1), with negativity for CK20, thyroglobulin, P40, and S-100 (Figure 2). These findings were compatible with a moderately differentiated adenocarcinoma metastatic to the brain. In conjunction with the morphological features and clinical-radiological findings, the immunohistochemical profile, particularly CK7 and TTF-1 positivity with CK20 negativity, favored a pulmonary origin; however, a primary pulmonary lesion was not definitively demonstrated.

**Figure 1:**
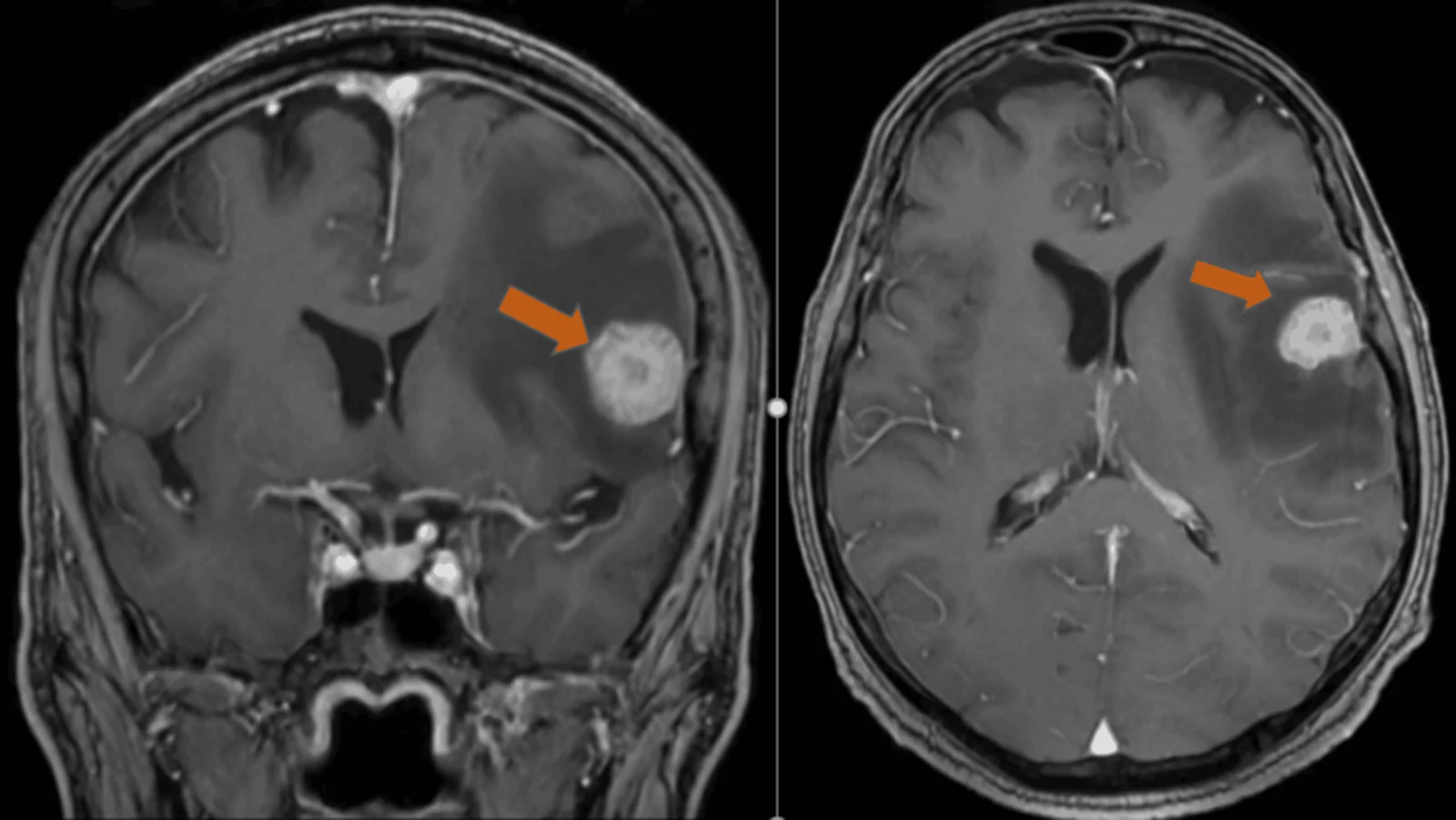
Contrast-enhanced T1-weighted brain MRI coronal (left) and axial (right) views demonstrating a 21-mm intra-axial cortico-subcortical lesion (arrow) in the left temporoparietal region with peripheral ring enhancement and mass effect on the ipsilateral lateral ventricle without midline shift.

**Figure 2:**
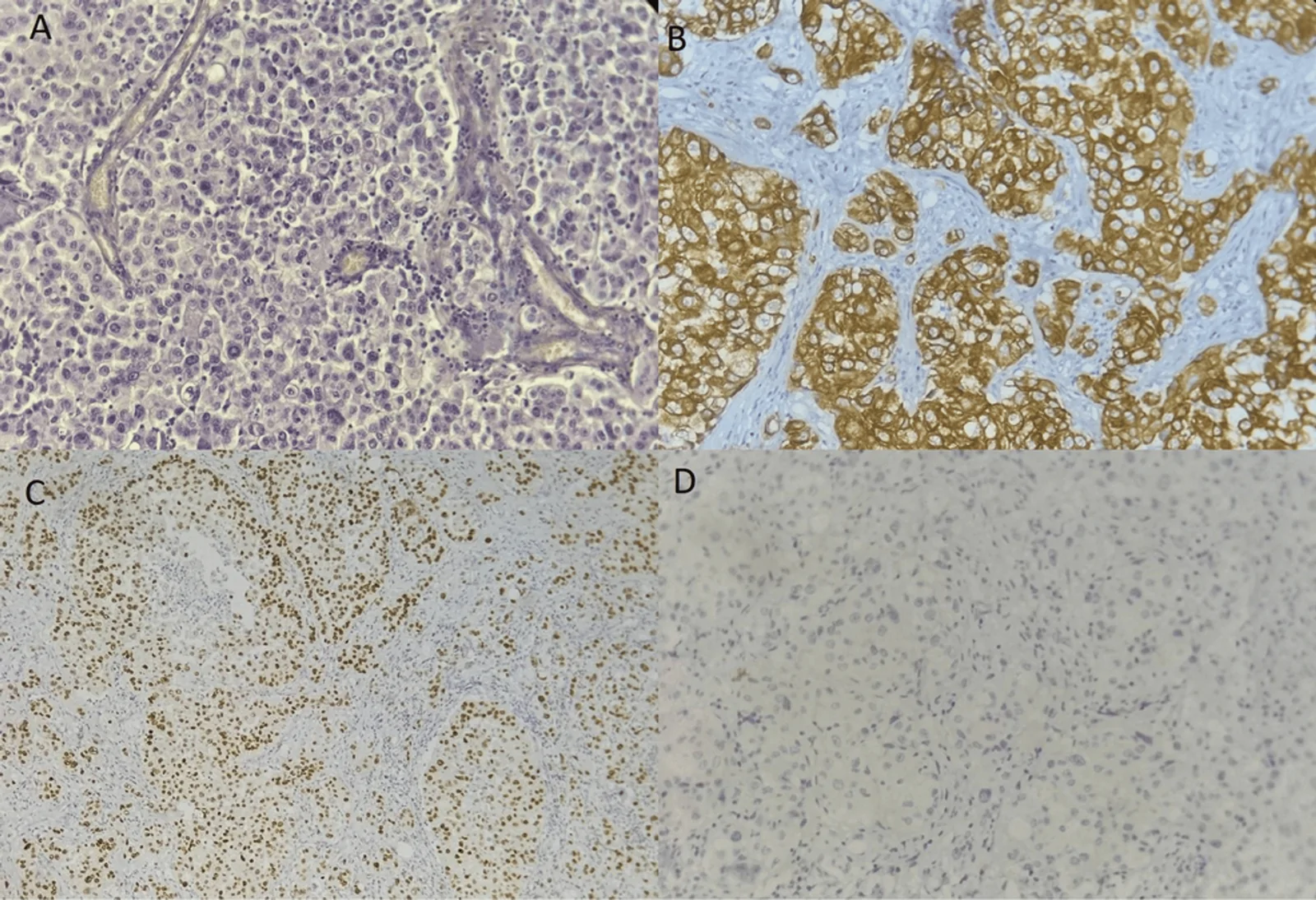
Histopathological and immunohistochemical features of the cerebral metastasis. (A) Hematoxylin-eosin-saffron (H&E) staining showing a cellular tumor composed of atypical epithelial cells arranged in nests consistent with a poorly to moderately differentiated carcinoma. (B) Tumor cells show strong, diffuse membranous positivity for CK7. (C) Tumor cells demonstrate diffuse nuclear positivity for TTF-1. (D) Tumor cells are negative for CK20. TTF-1: Thyroid transcription factor 1.

Molecular testing for actionable driver alterations and predictive biomarkers was not performed in this patient. The immunohistochemical analysis was used to characterize the tumor phenotype and support the suspected site of origin but was not considered a substitute for molecular profiling. 

A staging workup was requested to search for the primary lung tumor; as the CT scan returned normal findings, a PET scan was subsequently requested (Figure 3). PET-CT demonstrated hypermetabolic lymphadenopathy involving the right supraclavicular region (SUVmax 6), right inferior paratracheal region, right superior and inferior hilar regions, left para-aortic region (SUVmax 8), and subcarinal region (SUVmax 6.5). A questionable hypermetabolic focus was also noted in the mid-thoracic esophagus at the D6 level (SUVmax 4). No suspicious hypermetabolic lesions were identified in the remaining explored fields.

**Figure 3:**
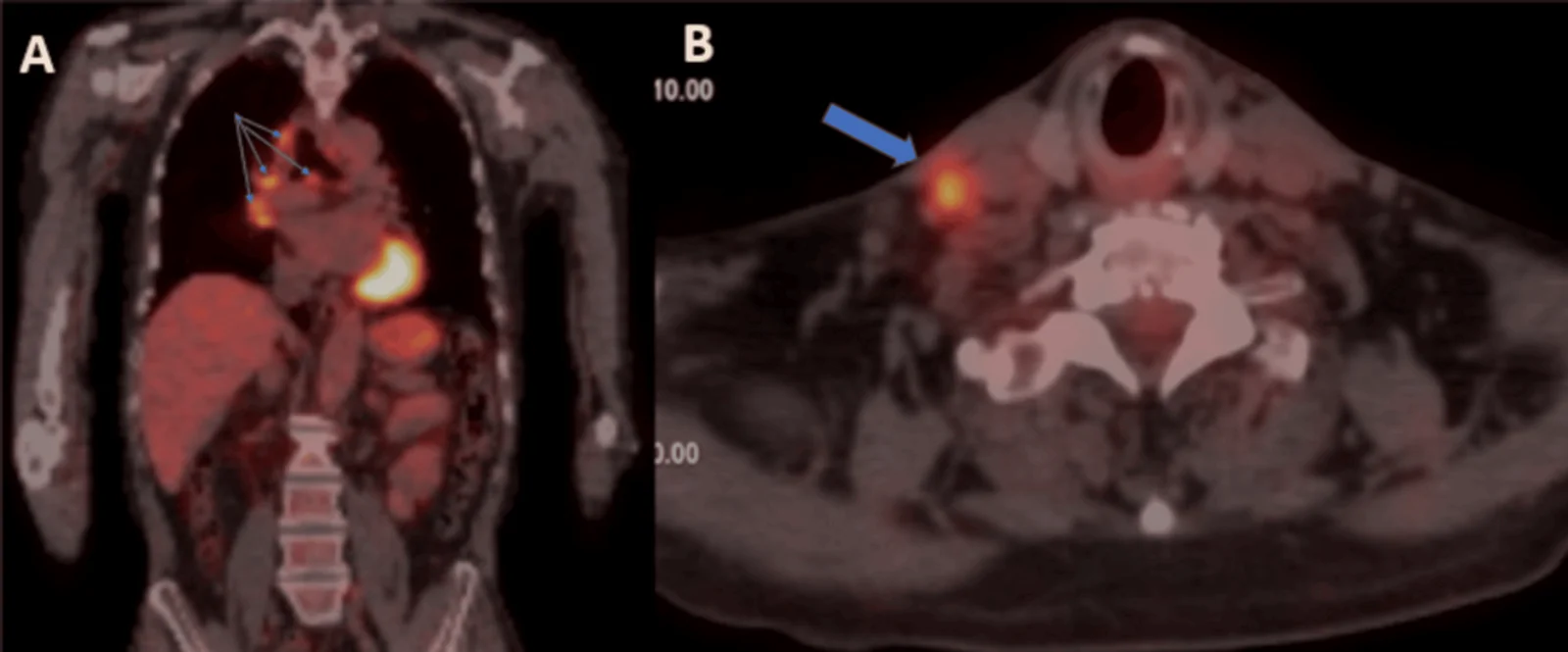
¹⁸F-FDG PET-CT coronal (A) and axial (B) fused images demonstrating hypermetabolic mediastinal hilar and right supraclavicular lymphadenopathy (arrow) without a definite hypermetabolic pulmonary lesion to account for a primary tumor. FDG: Fluorodeoxyglucose.

On endoscopy, upper GI mucosa appeared macroscopically normal, with a regular Z-line; the cardia was well negotiated at 42 cm from the dental arch. Erythematous fundal gastritis and erythematous-whitish antral gastritis were noted. The gastric mucosa appeared erythematous, and multiple biopsies were taken. Histopathological examination of the biopsy specimens showed no evidence of malignancy.

Following surgery, the patient underwent a session of stereotactic gamma knife radiotherapy, delivering a dose of 24 Gy in a single session to the resection bed of the cerebral metastasis. Subsequently, following multidisciplinary tumor board discussion, the decision was reached to initiate systemic chemotherapy with a cisplatin-vinorelbine regimen: cisplatin 80 mg/m² on day 1 and vinorelbine 80 mg/m² on days 1 and 8, repeated every 21 days for a planned total of three cycles. Response was to be evaluated by CT scan after completion of the cycles. The patient was informed of the treatment plan, its expected benefits, and its potential adverse effects, and gave informed consent.

## Discussion

Our case illustrates an unusual presentation of NSCLC in which a brain metastasis was the initial manifestation of disease. OP-NSCLC is characterized by histopathologic evidence of a pulmonary tumor obtained from nodal or metastatic tissue despite the absence of a radiographically or clinically identifiable primary lung lesion [6,7].

The rarity of occult NSCLC and the absence of a radiographically visible primary lesion complicate diagnosis, staging, and comparison across reported cases. Available population-level data remain limited, and the biology and natural history of this presentation are not yet well defined [5].

Published reports have described OP-NSCLC diagnosed from nodal or distant metastatic tissue despite the absence of a visible pulmonary primary [6-8]. In this setting, morphology and immunohistochemistry are central to assigning the most likely site of origin. A profile that includes TTF-1 or Napsin A positivity, usually with CK7 positivity and CK20 negativity, supports pulmonary adenocarcinoma when interpreted together with morphology and clinical findings [9]. This approach mirrors the profile observed in our patient (TTF-1 positive, CK7 positive, and CK20 negative). Other reports also show that NSCLC may first become apparent through symptoms caused by distant metastatic disease, including musculoskeletal pain [10]. Cross-sectional and functional imaging then define disease extent and guide the search for a primary site.

Together, this integrated approach enables diagnosis and treatment planning despite the absence of an identifiable primary lung lesion. Contrast-enhanced chest CT is the initial examination used to search for a pulmonary primary and to assess thoracic and distant disease. When CT is unrevealing, fluorodeoxyglucose PET/CT can refine staging, identify metabolically active sites, and help guide biopsy. A malignancy with a pulmonary phenotype but no visible primary should also be evaluated within a cancer-of-unknown-primary framework so that alternative primary sites are systematically excluded [11]. Management of OP-NSCLC presenting with a brain metastasis requires both local intracranial control and systemic disease management. Surgical resection is reasonable in selected patients, particularly for symptomatic lesions or those causing mass effect, and stereotactic radiosurgery to the surgical cavity is recommended for patients with limited resected brain metastases [12]. The surgery followed by stereotactic radiosurgery used in our patient was therefore consistent with contemporary brain-metastasis guidance. A similar presentation of brain metastasis from occult lung adenocarcinoma has also been reported [13]. When pulmonary origin is well supported, systemic treatment is generally extrapolated from advanced NSCLC management; the cisplatin-vinorelbine doublet used in this patient is among the platinum-based regimens historically used in metastatic NSCLC [14]. Treatment selection should ultimately be guided by metastatic extent, molecular findings, performance status, and access to targeted therapy or immunotherapy rather than by the absence of a detectable primary lesion alone.

A limitation of this case is the absence of molecular profiling for actionable driver alterations and predictive biomarkers. Such testing could have provided additional information relevant to therapeutic decision-making. Therefore, the immunohistochemical findings in this case should be interpreted as supporting the suspected pulmonary origin rather than as molecular confirmation of the primary site.

## Conclusions

This case highlights that cerebral metastasis can be the initial and sole manifestation of an occult malignancy with clinicopathological features favoring pulmonary origin, even without a radiographically identifiable primary pulmonary lesion. Clinicians should maintain a high index of suspicion for OP-NSCLC in patients presenting with brain metastases of unclear origin, and immunohistochemistry should be pursued early to establish tumor lineage when imaging fails to localize a primary site. A multimodal approach combining local control of the metastatic lesion with systemic therapy remains central to management, and molecular profiling should be incorporated whenever possible to guide treatment selection. Our patient’s stability following combined surgical, radiotherapeutic, and chemotherapeutic management supports the feasibility of this approach, though further studies are needed to define optimal long-term treatment strategies for this rare entity.

## References

[1] Bray F, Laversanne M, Sung H, Ferlay J, Siegel RL, Soerjomataram I, Jemal A (2024). Global cancer statistics 2022: GLOBOCAN estimates of incidence and mortality worldwide for 36 cancers in 185 countries. CA Cancer J Clin.

[2] Alduais Y, Zhang H, Fan F, Chen J, Chen B (2023). Non-small cell lung cancer (NSCLC): a review of risk factors, diagnosis, and treatment. Medicine (Baltimore).

[3] Wang S, Tang W, Jin F, Luo H, Yang H, Wang Y (2025). Comprehensive analysis of lung cancer metastasis: sites, rates, survival, and risk factors - a systematic review and meta-analysis. Clin Respir J.

[4] Waqar SN, Samson PP, Robinson CG (2018). Non-small-cell lung cancer with brain metastasis at presentation. Clin Lung Cancer.

[5] Cai J, Yang F, Wang X (2022). Occult non-small cell lung cancer: an underappreciated disease. J Clin Med.

[6] Lockwood M, Persaud J, Eldeiry D, Mina BA (2025). Occult primary non-small cell lung cancer without an identifiable primary lung tumor. Chest.

[7] Visneski S, Ferrari M, Waghchoure S (2025). Occult primary non-small cell carcinoma of the lung: a diagnostic challenge. American Journal of Respiratory and Critical Care Medicine.

[8] Woo FB, Chew SW, Huan NC, Tham JH (2026). Silent lungs, aching knee: Isolated knee pain as the initial presentation of lung adenocarcinoma. Univ Toronto Med J.

[9] Selves J, Long-Mira E, Mathieu MC, Rochaix P, Ilié M (2018). Immunohistochemistry for diagnosis of metastatic carcinomas of unknown primary site. Cancers (Basel).

[10] Ahmad H, Jannat H, Khan U, Ahmad N (2023). Unmasking non-small cell lung cancer: the unusual trail of hip pain and skeletal metastasis. Cureus.

[11] Greco FA (2024). Cancer of unknown primary site: a new era of practice-changing approaches to diagnosis, staging, and precision therapy. JCO Oncol Adv.

[12] Vogelbaum MA, Brown PD, Messersmith H (2022). Treatment for brain metastases: ASCO-SNO-ASTRO guideline. J Clin Oncol.

[13] Comitalo K, Abramian E, Taddonio M (2025). Atypical debut: unveiling brain metastasis from occult lung adenocarcinoma. American Journal of Respiratory and Critical Care Medicine.

[14] Reck M, Popat S, Reinmuth N, De Ruysscher D, Kerr KM, Peters S (2014). Metastatic non-small-cell lung cancer (NSCLC): ESMO Clinical Practice Guidelines for diagnosis, treatment and follow-up. Ann Oncol.

